# Potential Conflicts of Interest of Editorial Board Members from Five Leading Spine Journals

**DOI:** 10.1371/journal.pone.0127362

**Published:** 2015-06-04

**Authors:** Stein J. Janssen, Annelien L. Bredenoord, Wouter Dhert, Marinus de Kleuver, F. Cumhur Oner, Jorrit-Jan Verlaan

**Affiliations:** 1 Department of Orthopaedics, University Medical Center Utrecht, Utrecht, The Netherlands; 2 Department of Medical Humanities, Julius Center, University Medical Center, Utrecht, The Netherlands; 3 Department of Orthopaedics, VU University Medical Center, Amsterdam, The Netherlands; Rega Institute for Medical Research, BELGIUM

## Abstract

Conflicts of interest arising from ties between pharmaceutical industry and physicians are common and may bias research. The extent to which these ties exist among editorial board members of medical journals is not known. This study aims to determine the prevalence and financial magnitude of potential conflicts of interest among editorial board members of five leading spine journals. The editorial boards of: The Spine Journal; Spine; European Spine Journal; Journal of Neurosurgery: Spine; and Journal of Spinal Disorders & Techniques were extracted on January 2013 from the journals’ websites. Disclosure statements were retrieved from the 2013 disclosure index of the North American Spine Society; the program of the 20th International Meeting on Advanced Spine Techniques; the program of the 48th Annual Meeting of the Scoliosis Research Society; the program of the AOSpine global spine congress; the presentations of the 2013 Annual Eurospine meeting; and the disclosure index of the American Academy of Orthopaedic Surgeons. Names of the editorial board members were compared with the individuals who completed a disclosure for one of these indexes. Disclosures were extracted when full names matched. Two hundred and ten (29%) of the 716 identified editorial board members reported a potential conflict of interest and 154 (22%) reported nothing to disclose. The remaining 352 (49%) editorial board members had no disclosure statement listed for one of the indexes. Eighty-nine (42%) of the 210 editorial board members with a potential conflict of interest reported a financial relationship of more than $10,000 during the prior year. This finding confirms that potential conflicts of interest exist in editorial boards which might influence the peer review process and can result in bias. Academia and medical journals in particular should be aware of this and strive to improve transparency of the review process. We emphasize recommendations that contribute to achieving this goal.

## Introduction

The spinal surgery community has recently witnessed serious controversies and discussion concerning possible bias in scientific reports on the effects of a commercially available bone morphogenetic protein. This has compromised the standing of this community, and it was the direct reason for performing this study [1–4]. Financial and nonfinancial relationships between pharmaceutical or medical device industry, physicians, investigators, and academic institutions are common and generally considered essential for development of new technologies and advancement in medicine [5, 6]. However, these ties may at the same time create conflicts of interest: a set of circumstances that creates a risk that professional judgments or actions regarding a primary interest will be unduly influenced by a secondary interest [7]. Although conflict of interest policies typically focus on financial relationships, as these are relatively objective and quantifiable, all kinds of conflicts of interest exist including the desire for professional advancement, recognition for personal achievement, and favors to friends and colleagues [7]. Conflicts of interest arising from ties between industry and physicians may potentially bias research, influence medical decision-making and even jeopardize patient health and the public’s trust in medicine [3–5, 8–15]. As a result, reporting disclosures of (potential) conflicts of interest has become mandatory for authors of medical publications and presenters during scientific meetings to provide their audience with the opportunity to appreciate results in light of the disclosures, but this is not the case for reviewers and editors of scientific journals, nor for abstract reviewers of medical conferences [8]. Medical journals strive to be objective and reliable sources of scientific information within a specific field of medicine and go to great lengths to ensure scientific integrity of their publications. The editorial board members of medical journals are tasked with the duty to ensure integrity of the peer review process which is usually performed by internal or external reviewers [16, 17]. Editorial boards consist of the editor-in-chief, section or deputy editors, associate and assistant editors and an advisory board (herein referred to as editorial board members). The editor-in-chief is a journals’ editorial leader with final responsibility for publications and compliance with the policies of a journal; section or deputy editors manage the review process of submissions; advisory and associate editors assist editors in the peer review process and are often specialists in a specific field. Reviewers are often selected and approached by editors to review a manuscript; they are usually not paid and often have no formal relationship other than voluntary service as a reviewer.

Both editorial board members and reviewers are involved in editing the content of manuscripts and the decision to accept or reject the manuscript (often after revision following editor/reviewer suggestions) for publication. This process of editing of- and deciding on manuscripts can be influenced by numerous factors including the perspective of an editorial board member, degree of knowledge or experience with the topic under study and conflicts of interest [18–21]. Conclusions drawn in a manuscript pertaining the use of a medical device might for instance be adjusted, weakened or strengthened, or the manuscript itself could be rejected or accepted by editorial board members with (financial) ties to either the respective industry or industries producing competing devices. Authors from a manuscript describing positive results of a device from company A might, for example, receive more criticism by an editorial board member with financial ties to company B that produces competing devices compared to an editorial board member with no financial ties or an editorial board member with financial ties to company A [3, 4, 14, 22].

Although several publications have demonstrated the occurrence of bias in the peer review process as a result of conflicts of interest, it is not clear to what extent this exists in editorial boards [4, 14, 22]. This study aims to perform a first general assessment of (1) the prevalence of potential conflicts of interest among editorial board members of five leading spine journals, (2) the (financial) magnitude of these conflicts of interest, and (3) the relationships of editorial board members with the orthopedic/spinal implant device industry.

## Methods

### Retrieving basic information of members of editorial boards

**Fig 1:**
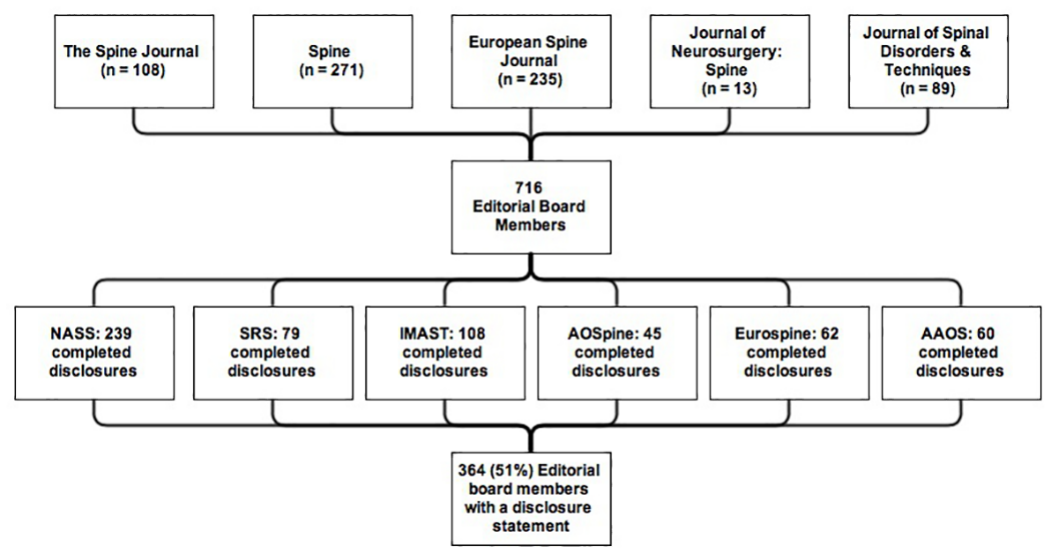
Flowchart demonstrating the number of editorial board members per journal and the number of participants that completed a disclosure per meeting or index. NASS = North American Spine Society, SRS = Scoliosis Research Society, IMAST = International Meeting on Advanced Spine Techniques, AOSpine = Arbeitsgemeinschaft für Osteosynthesefragen Spine, AAOS = American Academy of Orthopaedic Surgeons.

The full name and country of residence of every editorial board member of the following journals was extracted on January 2013 from the journals’ websites: *The Spine Journal; Spine; European Spine Journal; Journal of Neurosurgery*: *Spine; and Journal of Spinal Disorders & Techniques* [23–27]. These five journals are considered to be the leading journals for spine professionals based on their 2012 impact factor (Fig 1) [28]. To provide insight into the composition of the editorial boards of these journals, for every editorial board member the internet was searched for medical doctor (MD) and doctor of philosophy (PhD) degrees and primary employment function. Degrees and functions were extracted when both full name (i.e. last name, first name, and when available middle name/initial) and country of residence matched. Primary job functions were categorized into: spine surgeon (orthopedic surgeons; neurological surgeons; and general surgeons performing spinal surgery); other medical specialist; non-physician healthcare provider; researcher/investigator (including both clinical and fundamental research) and other. Degrees, functions, and countries of residence were presented as frequencies and percentages for editorial board members with complete data. All data used in this study is openly available online; therefore, approval by an institutional review board was not obtained.

### Identifying disclosure indexes

We retrieved disclosure statements from the 2013 annual disclosure index of the North American Spine Society (NASS; n = 4,066); the final program of the 20^th^ International Meeting on Advanced Spine Techniques (IMAST) in Vancouver, Canada (2013; n = 726); the final program of the 48^th^ Annual Meeting of the Scoliosis Research Society (SRS) in Lyon, France (2013; n = 863); the final program of the AOSpine global spine congress in Hong Kong, China (2013; n = 165); the posters and presentations of the 2013 Annual Eurospine meeting (n = 662); and the online disclosure index of the American Academy of Orthopaedic Surgeons (AAOS; number of individuals in the database not available) (Fig 1) [28–34].

These meetings and indexes were chosen because of the large number of participants, exclusive focus on spinal topics, and availability of disclosure statements. Disclosure statements were not available for: Canadian Spine Society; British Association of Spinal Surgeons; American Spinal Injury Association; Nordic Spinal Deformities Society; British Scoliosis Society; Brussels International Spine Symposium; and International Congress on Early Onset Scoliosis.

Disclosing for these meetings and indexes was mandatory for participants for reasons including (but not limited to): speaking engagements [29–33]; abstract and article authorships [29, 32, 34]; instructing [30–32]; managing or being able to control content of an activity [29–32, 34]; or official appointments to the office and representing a committee or board of the corresponding society [29, 32].

Disclosures considered to constitute a potential conflict of interest according to the respective societies included (but were not limited to) ownership, payments and reimbursements for: personal or professional services; stock or shareholder ownership; consulting (paid or unpaid); trips; travel; fellowship support; royalties; patents; gifts; grants and research support. Disclosures such as payments and reimbursements for governing boards of journals or societies and for medical publications were excluded, as these might be related to the function of editorial board member.

### Identifying potential conflicts of interest of editorial board members

The editorial offices of the five journals were contacted and asked to provide the disclosures of their editorial board members; only one office responded and stated that the disclosures were considered confidential. The Spine Journal lists disclosures of the executive editorial board on their website (9 editors) [35].

To determine whether editorial board members had a potential conflict of interest, we compared the full names of all editorial board members with the names of all individuals who completed a disclosure statement for the NASS, IMAST, SRS, AOSpine, or Eurospine and extracted the disclosures when full names matched [29–31, 33]. If multiple disclosures were found for an individual they were combined into one encompassing disclosure. Subsequently, the online disclosure index of the AAOS was searched for disclosure statements of editorial board members who did not disclose for the NASS, IMAST, SRS, AOSpine, or Eurospine meetings [29–33]. Disclosures were extracted from the AAOS index when full names matched and the disclosure statement was updated in 2012 or 2013.

The disclosures were categorized in: (1) potential conflict of interest (as specified in the previous paragraph), (2) nothing to disclose/no conflict reported (i.e. the editorial board member stated nothing to disclose), and (3) no disclosure statement listed for one of the meetings or indexes. The disclosure index of the NASS from 2013 contained the amount of financial relationship per disclosure [29]. Using this index; the potential conflict of interest group was categorized into: (a) more than $10,000 financial relationship reported, (b) less than or equal to $10,000 financial relationship reported, and (c) amount not disclosed [29]. Disclosures reporting a potential conflict of interest for the IMAST, SRS, AOSpine, Eurospine, or AAOS were classified as category (c) amount not disclosed, since the amount of financial relationship was not reported for these meetings or indexes [30–33].

The disclosure statements were searched for financial relationships with the six leading orthopedic/spinal medical device companies: Biomet, Smith and Nephew, Stryker, Zimmer, Medtronic, Johnson & Johnson, the latter including subsidiaries DePuy and Synthes [36].

## Results

### Editorial board members

A total of 716 editorial board members could be identified on the websites of the five spine journals representing 595 unique individuals (Table 1 & Fig 1). Ninety-eight (16%) individuals were members of more than one editorial board of these five spine journals (based on matching full name and country of residence). The editorial board members were from 41 countries in five continents; most members were from the North American continent (58%). The majority of the editorial board members were spine surgeons (75%).

**Table 1:** Baseline: editorial boards of the five leading spine journals.

	The Spine Journal (n = 108)†	Spine (n = 271)†	European Spine Journal (n = 235)†	Journal of Neurosurgery: Spine (n = 13)†	Journal of Spinal Disorders & Techniques (n = 89)†	Total Editorial Board Members (n = 716)	Total unique individuals (n = 595)
Impact Factor, 2012*	3.355	2.159	2.133	1.978	1.767	n/a	n/a
**Proportion of editorial board members appearing in at least on of the other spine journals**	50 (46%)	80 (30%)	36 (15%)	10 (77%)	43 (48%)	98 (14%)	98 (16%)
**Degree**	***n = 107‡***	***n = 270‡***	***n = 231‡***	***n = 13***	***n = 89***	***n = 710‡***	***n = 589‡***
Doctor of Medicine degree (MD)	92 (86%)	223 (83%)	191 (83%)	13 (100%)	86 (97%)	605 (85%)	499 (85%)
Doctor of Philosophy degree (PhD)	22 (21%)	87 (33%)	120 (52%)	1 (7.7%)	13 (15%)	243 (34%)	213 (36%)
**Continent of residence**	***n = 108***	***n = 271***	***n = 235***	***n = 13***	***n = 89***	***n = 716***	***n = 595***
North America	99 (92%)	194 (72%)	33 (14%)	12 (93%)	77 (87%)	415 (58%)	322 (54%)
Europe	0 (0%)	44 (16%)	174 (74%)	0 (0%)	5 (5.6%)	223 (31%)	207 (35%)
Asia	3 (2.8%)	25 (9.2%)	14 (6.0%)	1 (7.7%)	6 (6.7%)	49 (6.8%)	42 (7.1%)
Australia	1 (0.93%)	7 (2.6%)	5 (2.1%)	0 (0%)	1 (1.1%)	14 (2.0%)	13 (2.2%)
South America	5 (4.6%)	1 (0.37%)	6 (2.6%)	0 (0%)	0 (0%)	12 (1.7%)	8 (1.3%)
Africa	0 (0%)	0 (0%)	3 (1.3%)	0 (0%)	0 (0%)	3 (0.04%)	3 (0.50%)
**Primary job function**	***n = 100‡***	***n = 263‡***	***n = 219‡***	***n = 13***	***n = 88‡***	***n = 683‡***	***n = 564‡***
Spine surgeon	73 (73%)	186 (71%)	160 (73%)	13 (100%)	82 (93%)	514 (75%)	416 (74%)
Other medical specialist	14 (14%)	28 (11%)	16 (7.3%)	0 (0%)	3 (3.4%)	61 (8.9%)	56 (9.9%)
Non-physician healthcare provider	1 (1.0%)	12 (4.6%)	8 (3.7%)	0 (0%)	0 (0%)	21 (3.1%)	20 (3.5%)
Researcher/Investigator	12 (12%)	31 (12%)	34 (15%)	0 (0%)	3 (3.4%)	80 (12%)	65 (12%)
Other	0 (0%)	6 (2.3%)	1 (0.46%)	0 (0%)	0 (0%)	7 (1.0%)	7 (1.2%)

* Impact Factor for the year 2012 according to Journal Citation Reports, Thomson Reuters, 2013 [28].

^†^ Editorial board members as published on websites of the journals, January 2013 [23–27].

^‡^ only the traceable editorial board members are mentioned and used to calculate percentage per category

### Potential conflicts of interest

Two hundred and ten (29%) of the 716 editorial board members reported a potential conflict of interest and 154 (22%) reported nothing to disclose/no conflict reported, during the meetings in 2013 (Table 2). The remaining 352 (49%) editorial board members had no disclosure statement listed for one of the meetings or indexes; therefore, we were not able to trace the potential conflicts of interest of these individuals. Hence, more than half (58%, 210 of 364) of the traced editorial board members who had a disclosure listed for one of the meetings or indexes reported a potential conflict of interest. Potential conflicts of interest were not identifiable from the journals themselves, except for The Spine Journal which lists a disclosure of their executive editorial board on their website (9 editors).

**Table 2:** Potential conflicts of interest of editorial board members.

	The Spine Journal (n = 108)†	Spine (n = 271)†	European Spine Journal (n = 235)†	Journal of Neurosurgery: Spine (n = 13)†	Journal of Spinal Disorders & Techniques (n = 89)†	Total Editorial Board Members (n = 716)	Total unique individuals (n = 595)
**Potential conflict of interest**	41 (38%)	64 (24%)	46 (20%)	9 (69%)	50 (56%)	210 (29%)	156 (26%)
> 10,000$ financial relationship	18 (17%)	30 (11%)	9 (4%)	6 (46%)	26 (29%)	89 (12%)	64 (11%)
≤ 10,000$ financial relationship	10 (9%)	6 (2%)	8 (3%)	1 (8%)	6 (7%)	31 (4%)	22 (3.7%)
Financial relationship, amount not disclosed	13 (12%)	28 (10%)	29 (12%)	2 (15%)	18 (20%)	90 (13%)	70 (12%)
**Nothing to disclose/no conflict reported**	29 (27%)	67 (25%)	45 (19%)	0 (0%)	13 (15%)	154 (22%)	125 (21%)
Completed disclosure available*	70 (65%)	131 (48%)	91 (39%)	9 (69%)	63 (71%)	364 (51%)	281 (47%)
**Financial relationship disclosure with one of six leading medical device companies‡**	25 (23%)	48 (18%)	32 (14%)	9 (69%)	45 (51%)	159 (22%)	114 (19%)

* Editorial board members who completed a disclosure for: (1) North American Spine Society national spine meeting 2013 [29]; (2) Scoliosis Research Society 48th annual meeting & course 2013 [31]; (3) Scoliosis Research Society 20th International Meeting on Advanced Spine Techniques 2013 [30]; (4) AOSpine global spine congress 2013 [33]; (5) Eurospine 2013 Annual Meeting [34], and/or (6) the American Academy of Orthopaedic Surgeons 2012/2013 [32].

^†^ Editorial board members as published on websites of the journals, January 2013 [23–27].

^‡^ Biomet, Zimmer, Stryker, Smith & Nephew, Medtronic, Johnson & Johnson (including the subsidiaries DePuy & Synthes) [36].

The prevalence of reported potential conflicts of interest ranged from 20% for editorial board members of the European Spine Journal to 69% for editorial board members of the Journal of Neurosurgery: Spine. However, the percentage of retrieved disclosure statements of editorial board members varied largely; ranging from 39% for the European Spine Journal to 71% for the Journal of Spinal Disorders & Techniques. More than half of the traced editorial board members of every individual journal—except for Spine—reported a potential conflict of interest.

Eighty-nine (42%) of the 210 editorial board members with a potential conflict of interest reported a financial relationship of more than $10.000 (Table 2). The amount was not disclosed or not available per meeting program or index for 90 individuals that reported a financial relationship.

The majority, 159 of the 210 (76%) editorial board members that reported a potential conflict of interest had a financial relationship with one of the six leading medical device companies [36].

The potential conflicts of interest per region varied and was 64% (167 out of 259) for North American, 48% (38 out of 79) for European, 19% (4 out of 21) for Asian, 100% (1 out of 1) for Australian, and 0% (0 out of 4) for South American editorial board members with a disclosure listed for one of the meetings or indexes. The three editorial board members from Africa had no disclosures listed. However, the Asian (6.8%), Australian (2.0%), South American (1.7%), and African (0.04%) continents were quite underrepresented (totaling 11%).

## Discussion

Financial and nonfinancial relationships between industry and editorial board members generate a dilemma: although ties between pharmaceutical or medical device industry and the scientific journals are considered essential for sharing state-of-the-art scientific knowledge and moving the field of medicine forwards, these ties may at the same time create conflicts of interest, compromise the objectivity of the review process, and result in bias [4, 14, 22]. In this study we aimed to quantify the potential conflicts of interest of editorial board members of five leading spine journals. We found that 29% (210 of 716) of the editorial board members reported a potential conflict of interest at meetings and for indexes during the period 2012–2013, but this was not identifiable in the journals themselves. More than half (58%, 210 of 364) of the editorial board members for whom a disclosure statement was available for meetings or indexes declared an interest which might result in a potential conflicts of interest. More than one third of the editorial board members with a potential conflict of interest had a financial relationship of more than $10.000 during the prior calendar year. Most (76%, 159 of 210) of the conflicts of interest resulted from a (financial) relationship to medical device/implant companies. Obtaining information on potential conflicts of interest of editorial board members is cumbersome or impossible from journals’ websites. We therefore emphasize recommendations to make disclosure information generally available as part of a journals’ mission of transparent service to ensure that evidence is presented either without conflicts or at least with explicit conflicts that allow the reader to decide if the conflict is relevant.

This study has limitations, and the results should be interpreted with care. Our aim was to investigate whether we could find potential conflicts of interest but the methods we used cannot be considered comprehensive. Firstly, disclosure requirements varied among meetings and indexes and participants might have deemed their conflicts of interest irrelevant to disclose [37]. For example, a conflict of interest might have been relevant for the function as editorial board member but not with regard to the conference; this might have resulted in not reporting a relationship, as no conflict was perceived. Secondly, the role of editorial board members varies among journals and individuals; meaning that one editorial board member might have more influence in editing content than another. Potential conflicts of interest among editorial board members might therefore have different consequences. However, we were unable to account for this as the roles of editorial board members were not specified on the journals’ websites. Thirdly, the editorial boards published on the websites of the five spine journals might not include everyone involved in the review process as content from manuscripts may often require external expertise. This could have resulted in an underestimation of the size of the editorial boards and may have skewed our results. Fourthly, the editorial boards were extracted from the websites in January 2013 while the disclosure statements for the meetings and indexes were completed in the year 2012 and 2013; this could have resulted in disclosure statements that were not up-to-date. Fifthly, to match editorial board members to the completed disclosure statements of one of the meetings or indexes we compared surname, first name and, when available, middle name/initial; this could have led to mismatch due to other persons sharing the same name. However, this is unlikely to have occurred within the relatively small community of spinal professionals. Sixthly, as demonstrated by Jegede et al. (2011) instructions for completing a disclosure are not always clear and may be prone to potential errors and inconsistencies [12, 38]. In the present study 51% of the editorial board members had a disclosure statement listed for one of the meetings or indexes; no disclosure statement was found for the remaining 49%. Underdisclosure is, therefore, more likely to have occurred than overdisclosure; the magnitude of potential conflicts of interest among editorial board members (29%) found in this study is, as a result, probably a conservative estimate [39].

The question arises whether this existence of potential conflicts of interest among editorial board members is confined to the field of spine surgery. The journals with the highest impact factors in the field of cardiology and general medicine were screened for online availability of editorial board disclosures. Plos Medicine, the Annals of Internal Medicine and the Journal of the American College of Cardiology are among the highest ranked journals within their field and have online disclosures listed of their executive editorial boards. Disclosures of other editorial board members (i.e. associate, advisory, or assistant editorial board members) are not listed on the websites of these journals. Four out of 10 editors in Plos Medicine reported a potential conflict of interest (40%), 3 out of 7 editors in Annals of Internal Medicine reported a potential conflict of interest (43%), and 15 out of 17 editors in the Journal of the American College of Cardiology (88%) reported a potential conflict of interest [40–42]. Furthermore, a study among clinical medical journal editors by Wong et al. demonstrated that 13% of responding editors (N = 11, 95% confidence interval: 7 to 21) received industry funding as salary income or directly in support of research [21]. These findings indicate that potential conflicts of interest do not only exist among editorial boards in spine journals; all fields of medicine are probably confronted, more or less, with conflicts of interest of editors.

In this study we have focused on the conflict of interest based on financial relationships of editorial board members. We have not studied other potential conflicts, such as between competing researchers within a field. It is possible and even likely that reviewer/editor and author are competing in the same research field where first publications, citations and grant funding is very competitive. This might bias the editor or reviewer in the peer review process, leading to delayed publication or rejected manuscripts.

Conflict of interest of editorial board members has gained substantial attention over the past 10 years. Haivas et al. (2004) surveyed the senior editors of 30 general medicine journals and demonstrated that 19 (63%) felt it was important to declare the financial conflicts of interest of their editors; 13 (43%) felt that this was important for all of their editorial board members and 11 (37%) for other editorial advisers [43]. Editors’ associations including the Committee on Publication Ethics (COPE), World Association of Medical Editors (WAME), International Committee of Medical Journal Editors (ICMJE), Council of Science Editors (CSE), and American Medical Association (AMA) formulated guidelines on how to handle conflict of interest disclosures of editors to ensure objectivity and decrease the chance of bias in the review process [44–48]. However, a recently published study by Bosch et al. (2013) demonstrated that among 399 high-impact biomedical journals only 40% required a disclosure of conflict of interest from their editors [49]. Wong et al., demonstrated that 60% of editors report that their journals collect data on whether editorial board members have industry funding sources, and 63% of these journals publicly report these data [21]. We found that only one of the five spine journals publicly reported disclosures of their executive editorial board.

We found that a substantial percentage (29%) of editorial board members in spine journals have a potential conflict of interest, which is, as said, probably an underestimation since we were only able to trace about half of all editorial board members. This finding confirms that potential conflicts of interest exist within editorial boards which might subsequently influence the peer review process and may result in bias, potentially leading to altered conclusions or outright rejection of submitted manuscripts. Academia and medical journals in particular should be aware of this potential bias and strive to improve transparency of the review process. We therefore emphasize the following recommendations as proposed by the COPE, WAME, ICMJE, CSE, and AMA [44–48, 50, 51].

These committees recommend that: (1) medical journals should have explicit conflict of interest policies for their editorial board and reviewers, which should be available online, and (2) medical journals should publicly report current and past relationships of editors and reviewers that could lead to conflicts of interest, including the type of relationship (e.g. grants, consultancy, stock ownership), dollar amounts, to give readers the opportunity to appreciate the results bearing in mind the disclosures of the editors and reviewers involved in the review process [44–46, 49]. For journals using a blinded peer review system this could be done while still hiding the identity of reviewers and editors by: (1) providing the reader with the anonymized conflicts of interest, or (2) having an external journal auditor check these potential conflicts of interest. The disclosures of reviewers and editors involved in rejecting manuscripts could be tracked in a database to make the review process fully transparent, again hiding the identity of reviewers and editors. In addition to increasing transparency, journals should aim to minimize bias in the review process. This can be facilitated by the introduction of “Evidence and Methods” deputy editors with no relationships to device or pharmaceutical companies, reviewing the methodological rigor and proper reporting of error and potential bias as proposed by Carragee et al. [50]. Another suggestion is the preclusion of peer reviewers and editors with a conflict of interest related to the study under review; however, this might be difficult due to the expertise of some editors [50]. One of the five included spine journals (Journal of Spinal Disorders & Techniques) recently started following the recommendations proposed by the ICMJE. We recommend other journals to adopt these guidelines as well.

Importantly, one should be aware that disclosure is not a panacea: it may result in a situation that puts more weight on the biased information [51]. When an editor declares a conflict of interest, the readers might assess the articles in a journal as more reliable [51]. Financial and nonfinancial relationships between industry and editorial board members are not necessarily unethical but at least full transparency and awareness are necessary to maintain research integrity and public trust.

**Recommendations to improve transparency of the review process [44–50]**

- Journals should have explicit conflict of interest policies for editorial board members and reviewers, which should be available online
- Potential conflicts of interest of individuals involved in the editorial and review process should be reported
- A regularly updated database including disclosures of editors and reviewers should be published
- Journals should aim to minimize bias in the review process. This can be facilitated by: (1) the introduction of “Evidence and Methods” deputy editors with no relationships to device or pharmaceutical companies, reviewing the methodological rigor and proper reporting of error and potential bias, (2) preclusion of conflicted peer reviewers and editors where possible
